# Predictors of social support, physical health and mental health among food insecure internally displaced persons in Turkana, Kenya

**DOI:** 10.1186/s13031-020-00303-y

**Published:** 2020-08-07

**Authors:** Catherine Gichunge, Daniel Mutiso, Jenny Brynjarsdottir

**Affiliations:** 1 School of Nursing and Public Health, Chuka University, Chuka, Kenya; 2 School of Science and Technology, Turkana University College, Turkana, Kenya; 3 Department of Mathematics, Applied Mathematics, and Statistics, Case Western Reserve University, Cleveland, USA; 4 Faculty of Physical Sciences, University of Iceland, Reykjavik, Iceland

**Keywords:** Internally displaced persons, Food insecurity, Mental health, Social support, Physical health, Turkana, Kenya

## Abstract

**Background:**

Food insecurity and the mental and physical health of internally displaced persons (IDPs) is a public health concern. The aim of this study was to determine the predictors of social support, physical and mental health among food insecure IDPs in Nakwamekwei IDP camp in Turkana, Kenya.

**Methods:**

A cross sectional study was conducted among 159 household heads living in the camp. Analysis was conducted using statistical summaries, logistic regression and linear regression.

**Results:**

Ninety four percent (94%) of the households were severely food insecure and the rest of the households had moderate food insecurity. Majority of the household heads (77%) had symptoms of depression and those with five or more children were 3 times (95%CI, 1.31–9.24; *p* = 0.015) more likely to be have symptoms of depression, while those who were married were less likely to have the same (95%CI, 0.14–0.92; *p* = 0.038). Seventy six percent (76%) of the household heads had anxiety symptoms; none of the predictor variables were significantly associated with anxiety symptoms in the adjusted analysis. Those who had spent many years in the camp, were older, and had more children had significantly poorer physical health (*p* = 0.042, *p* = 0.001, and *p* = 0.047, respectively). Those who were married and those who had experienced violence in the current camp had significantly higher social support (*p* = 0.001 and *p* = 0.006, respectively).

**Conclusions:**

Participants have been living in camp for the last 10 years hence the need to improve their living conditions, address their physical and mental health as well as food insecurity. This can be done by providing the participants with safe drinking water, constructing pit latrines to prevent communicable disease and adhere to the Sphere recommendations for sanitation and hygiene as well as training them in income generating activities to mitigate the high unemployment and food insecurity rates. The IDPs should be integrated into the local community to bring an end to their protracted displacement.

## Background

Kenya has witnessed violence in every national election since the introduction of multipartism [1]. The post-election violence that occurred after the disputed general elections in 2007 reported the highest fatalities, loss of property and displacement [2]. Unfortunately majority of the displaced persons are still living in camps for internally displaced persons (IDPs) [2, 3].

The displacement and relocation that IDPs go through may lead to weakening or loss of the social support that they had. Social support is the perception or experience that one is loved, cared for, esteemed and valued by others and part of a social network of mutual assistance [4]. This support can come from family, friends, neighbours or organizations in the community. IDPs have also been found to suffer from food insecurity [5] and poor physical health [6]. Little is known regarding the relationship between food security social support, mental health and physical health among IDPs.

The aim of this study was to examine the predictors of social support, physical and mental health among IDPs living in Nakwamekwi IDP Camp, Turkana County.

## Methods

This study used a cross sectional survey. The study was conducted in Nakwamekwi IDP Camp, established in 2008 when people displaced from other volatile regions in the country following the 2007–2008 post-election violence moved backed to Turkana, their original home. Systemic random sampling was used to obtain a sample of household heads from a total of 400 households. To ensure randomization in choosing the first participant (i), participants of the first and second household were made to pick one piece of paper with the number after which the second household was selected. Therefore, the systematic sample consisted of participants in the series; i*, i + l, i + 2 l........., i + (n-1) l.*

*Where:*

*i = the first participant*

*l = the sampling interval*

*n = the sample*

In this study, a household was defined as a group of people living in the same dwelling compound within the camp while the household head was defined as the person, male or female who was responsible for the general welfare of the other members living in the same household. Only the household head was interviewed as they were better placed to provide an accurate account of the household situation as well as save time and the cost of the survey. A high number of women household heads were sampled in this study supporting findings that women outnumber men in IDP populations [7, 8].

A researcher-administered questionnaire was used to collect data. Mental health was assessed using the Hopkins Symptom Checklist (HSCL) which consists of 25 items that assess symptoms of anxiety (10 items) depression (15 items). Those with scores of > 1.75 for each subscale are considered to have depression and anxiety symptoms. This scale has been used among displaced populations [9, 10]. Physical health was measured using the 7-item short version of the 26 item WHO Quality of Life-BREF (WHOQOL-BREF) tool that has been used in Kenya before [11] and higher scores indicate better physical health while lower scores indicate poor physical health. Social support was measured using the Medical Outcomes Study Social Support Survey (MOS-SSS). This tool contains 19 items which measures tangible support, affectionate support, emotional support and positive social interaction. The MOS-SSS was scored following set guidelines from the authors and higher scores indicate more social support [12]. The Household Food Insecurity Access Scale (HFIAS) was used to measure household food security. The HFIAS consists of nine questions and the scores range from 0 to 27 with lower scores indicating severe food insecurity. Following HFIAS guidelines [13] the households were categorized as food secure or food insecure. Food insecure households were further categorized as having mild food insecurity, moderate food insecurity and severe food insecurity. The HFIAS has been used and validated in several countries [13] and used among various conflict affected population [5].

Data were first entered into Statistical Package for the Social Sciences (SPSS) version 20.0 and thereafter loaded into R [14] for further analysis. The internal consistency of the scales used were: food security (α = 0.86), mental health (α = 0.89: anxiety symptoms subscale α = 0.88, depression symptoms subscale α = 0.84), physical health (α = 0.73) and social support (α = 0.92). Frequencies, means, and standard deviations were calculated for all the variables. Logistic regression was used to determine predictors of mental health and unadjusted (one variable at a time) and adjusted (all variables in one model) odds ratios were obtained. Multiple linear regression analysis was used to determine predictors of social support and physical health. A value of *p* < 0.05 was used to determine statistical significance. Ethical approval was provided by Mount Kenya University Ethical Review Committee (MKU/ERC/0094). Participants provided oral informed consent while confidentiality and anonymity was maintained.

## Results

**Fig. 1:**
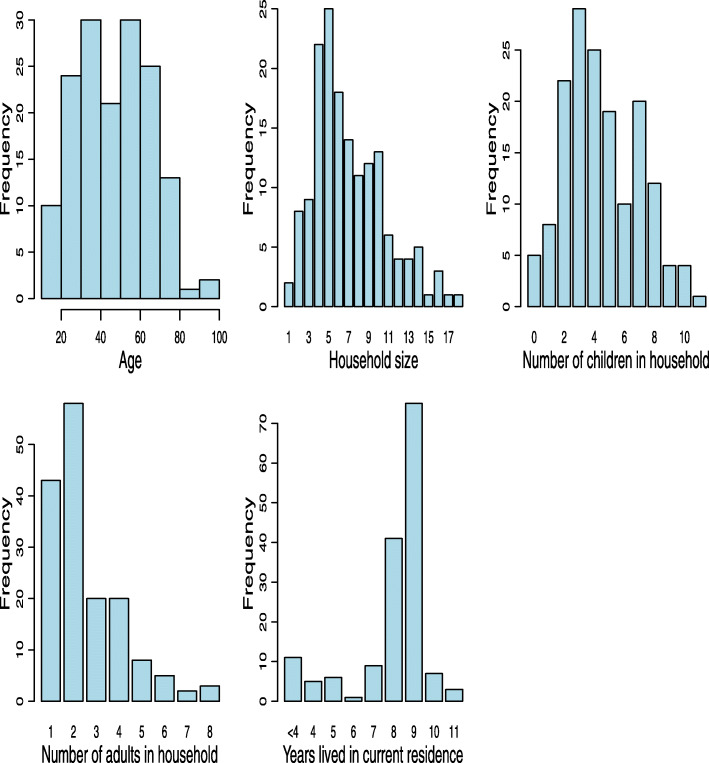
Histograms of quantitative demographic characteristics

A total of 159 household heads, also referred to as participants, were interviewed (response rate of 79.1%). The 159 households comprised of 1059 members (M = 6.66, SD ± 3.17) of which 717 (67.70%) were children aged 18 years and below. The mean age of the participants was 47.8 (SD ± 18.5, range 18–92) and 115 (72.3%) were female. The participants had lived in the camp for an average of 7.9 (SD ± 2.2, range 0.3–11) years, 92 (57.9%) were married, almost all had primary education and below (*n* = 152, 95.6%), while 155 (97.5%) were unemployed. All participants were food insecure with almost all the households experiencing severe food insecurity (*n* = 149, 93.7%) and none had access to a toilet or protected water source. A summary of participants’ characteristics is outlined in Table 1 and histograms of non-categorical variables are shown in Fig. 1 (Online Resource 1).

**Table 1:** Demographic characteristics

Characteristic ***(N = 159)***	Mean (SD) / N (%)
Age	47.8 (18.5)
Household size	7.1 (3.6)
Children in household	4.5 (2.5)
Adults in household	2.6 (1.6)
Years lived in current residence	7.9 (2.2)
Gender	
Female	115 (72.3%)
Male	44 (27.7%)
Marital status	
Single	67 (42.1%)
Married	92 (57.9%)
Employment status	
Employed	4 (2.5%)
Unemployed	155 (97.5%)
Education	
Primary education and below	152 (95.6%)
High school education and above	7 (4.4%)
Experienced violence in current residence (camp)	
No Answer	1
Yes	42 (26.6%)
No	116 (73.4%)
Lost family members during displacement	
No Answer	1
Yes	86 (54.4%)
No	72 (45.6%)
Food insecurity	
Food Secure	0 (0.0%)
Mild Food Insecurity	1 (0.6%)
Moderate Food Insecurity	9 (5.7%)
Severe Food Insecurity	149 (93.7%)
With depression symptoms	
Yes	123 (77.4)
No	36 (22.6)
With anxiety symptoms	
Yes	121 (76.1)
No	38 (23.9)

More than three quarters (*n* = 123, 77.4%) of the household heads had depression symptoms. In the adjusted analysis (see Table 2), married household heads were less likely to have depression symptoms compared to the unmarried (AOR = 0.37; 95%CI, 0.14–0.92; *p* = 0.038). Household heads living in households with five or more children were 3.32 times more likely have depression symptoms compared with those living in households with less than five children (AOR = 3.32; 95%CI, 1.31–9.24; *p* = 0.015). Those who had lived in the camp for more than 9 years were 0.24 times more likely have depression symptoms (AOR = 0.24; 95%CI, 0.09–0.59; *p* = 0.003). Gender, education level, age and number of adults in household were not associated with the risk having depression symptoms (Table 2).

**Table 2:** Logistic regression analysis for predictors of depression^a^ among IDPs in Turkana Kenya

Predictors	AOR	CI	***p***-val	UOR	CI	***p***-val
**(*N = 159*)**						
**Gender: Male**	0.83	0.30–2.30	0.708	0.83	0.38–1.93	0.661
**Marital status: Married**	0.37	0.14–0.92	**0.038**	0.45	0.19–0.98	0.051
**Education: High school education and above**	4.25	0.55–92.80	0.230	1.79	0.29–34.50	0.594
**Years lived in current residence: 9–12**	0.24	0.09–0.59	**0.003**	0.30	0.13–0.67	**0.005**
**Age: 41–70**	2.36	0.90–6.52	0.086	2.69	1.19–6.33	0.020
**Age: 71–100**	0.97	0.26–3.96	0.962	1.00	0.32–3.52	1.000
**Adults in household: 6–15**	0.40	0.07–3.27	0.335	1.18	0.28–8.08	0.837
**Children in household: 5–11**	3.32	1.31–9.24	**0.015**	2.48	1.13–5.79	**0.028**
**Experienced violence in current residence (camp): No**	1.04	0.39–2.65	0.929	1.08	0.45–2.44	0.853
**Lost family members during displacement: No**	0.99	0.40–2.48	0.976	0.92	0.43–1.95	0.821

^a^Hopkins Symptom Checklist (HSCL); cut off points>1.75 symptoms of depression

Seventy six percent (*n* = 121, 76.1%) of the participants had anxiety symptoms. None of the variables were significantly associated with anxiety symptoms in the adjusted analysis (Table 3). However, there was a slight (but not significant) increase in anxiety symptoms with an increase in number children in household as well as the age group of 41–70 years.

**Table 3:** Logistic regression analysis for predictors of anxiety^a^ among IDPs in Turkana Kenya

Predictors	AOR	CI	***p***-val	UOR	CI	***p***-val
**(*N = 159*)**						
**Gender: Male**	0.47	0.18–1.19	0.109	0.66	0.30–1.48	0.304
**Marital status: Married**	0.63	0.27–1.46	0.290	0.55	0.25–1.18	0.134
**Education: High school education and above**	1.36	0.25–10.69	0.740	0.78	0.16–5.58	0.767
**Years lived in current residence: 9–12**	0.58	0.25–1.31	0.198	0.60	0.28–1.26	0.186
**Age: 41–70**	2.09	0.84–5.41	0.118	2.01	0.92–4.48	0.081
**Age: 71–100**	1.80	0.47–8.23	0.410	1.36	0.42–5.35	0.626
**Children in household: 5–11**	2.10	0.92–4.99	0.084	1.99	0.94–4.44	0.079
**Experienced violence in current residence (camp): No**	1.66	0.69–3.92	0.249	1.72	0.76–3.77	0.181
**Lost family members during displacement: No**	1.37	0.58–3.34	0.482	1.13	0.54–2.40	0.745

^a^Hopkins Symptoms Checklist (HSCL); cut off points > 1.75 symptoms of anxiety

Participants tended to have poorer physical health if they had spent more years in the camp (− 1.41; 95%CI, − 2.76- -0.04; *p* = 0.042), were older (− 0.28; 95%CI,-0.44- -0.12; *p* = 0.001), and had more children in their households (− 1.38; 95%CI, − 2.75- -0.02; *p* = 0.047), see Table 4. Social support was significantly associated with marriage and experience of violence in the camp. Those who were married had significantly higher social support (OR = 8.16, 95%CI, 3.22–13.10; *p* = 0.001), and those who had not experienced violence in the camp had significantly less social support (R = -7.69, 95%CI,-13.09- -2.28; *p* = 0.006), see Table 4.

**Table 4:** Multiple linear regression analysis for predictors of physical and social health among IDPs in Turkana Kenya

	Physical health*			Social health**		
***Predictors***	***Estimates***	***CI***	***p***	***Estimates***	***CI***	***p***
**(*N = 159*)**						
**Gender: Male**	−0.25	−6.72 – 6.22	0.940	1.29	−4.17 – 6.76	0.641
**Marital status: Married**	0.50	−5.35 – 6.34	0.867	8.16 ^**^	3.22–13.10	**0.001**
**Education: High schooleducation and above**	5.50	−8.04 – 19.05	0.423	2.30	−9.14 – 13.74	0.692
**Years lived in currentresidence**	−1.41 ^*^	−2.76 – −0.05	**0.042**	− 0.10	−1.25 – 1.04	0.863
**Age**	−0.28 ^***^	−0.44 – − 0.12	**0.001**	−0.04	− 0.18 – 0.09	0.540
**Adults in household**	−0.37	−2.49 – 1.76	0.732	−0.17	−1.96 – 1.63	0.855
**Children in household**	−1.38 ^*^	−2.75 – −0.02	**0.047**	0.21	−0.95 – 1.36	0.722
**Experienced violence incurrent residence (camp): No**	3.12	−3.28 – 9.52	0.336	−7.69 ^**^	−13.09 – −2.28	**0.006**
**Lost family membersduring displacement: No**	0.10	−5.81 – 6.01	0.974	4.90	−0.09 – 9.89	0.054
**Observations**	155			155		
**R**^**2**^**/ R**^**2**^**adjusted**	0.200 / 0.151			0.181 / 0.130		

*p < 0.05 ** p < 0.01 *** p < 0.001

*WHOQOL-BREF (WHO Quality of Life-BREF); higher scores indicate better physical health

**Medical Outcomes Study Social Support Survey; higher scores indicate more social support

## Discussion

The health of IDPs is affected by displacement [15, 16]. The overall prevalence of depression and anxiety symptoms in this study are consistent with findings from other studies conducted among IDPs [6, 10, 11, 16, 17]. These high levels of depression and anxiety symptoms in this study may be attributed to the high prevalence of household food insecurity that may have caused worry and uncertainty among the household heads [18, 19]. Majority of the participants have been in the IDP camp for more than 5 years and long stay in camps has been associated with poor mental health among conflict afflicted populations [6, 15]. However married participants were less likely to have symptoms of depression supporting evidence marriage promotes mental health [6, 20, 21].

Older participants had poor health and these results are supported by other studies on IDPs [22]. Longer duration in the IDP camp was also associated with poor health [11, 17]. Participants in households with many adults and children were more likely to have poor health. IDP camps are usually overcrowded [23, 24] and this particular camp in Turkana is no exception and crowded conditions negatively affects health by facilitating spread of communicable diseases [23, 24]. High unemployment rates may also have contributed to the poor physical health. High food insecurity levels, lack of toilets and protected water sources may have contributed to participants’ poor health, as poor sanitation and food insecurity is a major contributor of poor health among conflict afflicted populations [6, 24].

Marriage is seen as offering social support, an important component of social health hence the high levels of social support among the married participants [20, 25]. Participants who had lost family members during displacement experienced violence in the camp, findings that show family members are a source of social support and people with low social support are more vulnerable to victimization and violence [26, 27].

Some limitations should be considered when interpreting this study’s results. First, due to lack of funds only one camp was visited and the sample size was also limited. Thus the results may vary with those from other IDP camps in the country as well as explain why few predictors were statistically significant. Second the measures used to measure depression and anxiety are for screening possible symptoms and not diagnostic. Third, poor specificity of the mental health measure may have resulted to a high percentage of participants indicating depression and anxiety symptoms.

## Conclusion

The National and County governments should consider resettling the IDPs who have overstayed in the camp in order to improve their physical, mental and social wellbeing. Meanwhile, provision of food, safe drinking water and toilets should be done to mitigate food insecurity and improve the health of the IDPs in the camp.

## Data Availability

The datasets used and/or analyzed during the current study are available from the corresponding author on reasonable request.

## Abbreviations

IDP: Internally Displaced Persons
AOR: Adjusted odds ratio
UOR: Unadjusted odds ratio
